# EGFR and K-Ras mutations in women with lung adenocarcinoma: implications for treatment strategy definition

**DOI:** 10.1186/s13046-014-0077-6

**Published:** 2014-10-11

**Authors:** Virginia Rotella, Lorenzo Fornaro, Enrico Vasile, Carmelo Tibaldi, Laura Boldrini, Antonio Chella, Armida D’Incecco, Giovanna Cirigliano, Aldo Chioni, Cristiana Lupi, Elisa Sensi, Laura Ginocchi, Simona Giovannelli, Maria Cristina Pennucci, Gabriella Fontanini, Editta Baldini

**Affiliations:** U.O. Oncologia Medica, Ospedale San Luca, Lucca, Italy; U.O. Oncologia Medica 2 Universitaria, Azienda Ospedaliero-Universitaria Pisana, Pisa, Italy; U.O. Oncologia Medica, Ospedale Civile, Livorno, Italy; Dipartimento di Patologia Chirurgica, Medica, Molecolare e di Area Critica, Università di Pisa, Pisa, Italy; U.O. Pneumologia, Azienda Ospedaliero-Universitaria Pisana, Pisa, Italy; U.O. Oncologia Medica, Ospedale della Misericordia, Grosseto, Italy; U.O. Anatomia Patologica III, Azienda Ospedaliero-Universitaria Pisana, Pisa, Italy; U.O. Oncologia Medica, Ospedale di Carrara, Carrara, Italy

**Keywords:** Lung adenocarcinoma, EGFR, K-Ras, Chemotherapy, Tyrosine-kinase inhibitors

## Abstract

**Background:**

We aimed at investigating the outcomes of female patients with stage IIIB-IV adenocarcinoma of the lung according to EGFR and K-Ras mutational status.

**Methods:**

One hundred and three consecutive female patients genotyped at a single Italian Institution were analyzed. Patients were planned to receive first-line platinum-based chemotherapy (CT) and a salvage treatment with anti-EGFR tyrosine-kinase inhibitors (TKIs) was proposed irrespective of tumor mutational status. EGFR (exons 18–21) and K-Ras (exon 2, codons 12–13) mutations were evaluated by real-time PCR and pyrosequencing. The association of mutational status with clinical variables and treatment benefit was investigated by chi-square test and log-rank test.

**Results:**

EGFR and K-Ras mutations were found in 31 (30%) and 13 (15%) cases, respectively. Sixty-six patients received platinum CT: no correlation was observed between EGFR or K-Ras mutational status and response rate (RR) (p > 0.05). However, patients treated with first-line CT harboring EGFR activating mutations experienced a significantly reduced progression-free survival (PFS) in comparison with wild-type ones (4.4 vs. 6.4 months, respectively; HR 0.597, 95% CI 0.287-0.975; p = 0.048). Thirty-nine patients received salvage treatment with erlotinib: EGFR activating mutations were significantly correlated with RR (60% vs. 12.5%; p = 0.004) and PFS (11.4 vs. 4.5 months; HR 0.491, 95% CI 0.216-0.936; p = 0.044). Responses to erlotinib were not reported among women with K-Ras mutant tumors, while 50% of those with wild-type K-Ras achieved an objective remission (p = 0.296). Median PFS (3.5 vs. 8.8 months; HR 0.284, 95% CI 0.015-0.510; p = 0.010) and OS (3.9 vs. 19.8 months; HR 0.158, 95% CI 0.001-0.075; p < 0.001) were significantly shorter among K-Ras mutant patients treated with TKI.

**Conclusions:**

In our population of Caucasian women with advanced lung adenocarcinoma we observed that the presence of EGFR activating mutations correlates with a significant reduction in the benefit from first-line platinum-based CT, emphasizing the importance of an upfront use of anti-EGFR TKIs in this patient subset. K-Ras mutations seem to correlate with a detrimental effect from anti-EGFR TKI, but this finding deserves further investigation.

## Background

The epidermal growth factor receptor (EGFR) and K-Ras are among the most investigated molecular drivers in solid tumors. In particular, EGFR is addressed as an interesting target for several biologic agents, mainly monoclonal antibodies (acting at the cellular surface level) and small molecules (inhibiting the intracellular tyrosine-kinase domain activity) [1]. EGFR overexpression often correlates with a worse prognosis is several tumors [2] and the blockade of the EGFR-driven cascade has been proved successful in non-small cell lung cancer (NSCLC), colorectal, head and neck and breast cancer [1]. Mutations in K-Ras, occurring at different levels along the gene sequence, have been described in multiple malignancies [3]. Moreover, K-Ras has been the first predictive biomarker identified and routinely used in advanced colorectal cancer management: mutations in this key oncogene predict the benefit from the anti-EGFR monoclonal antibodies panitumumab and cetuximab [4], and the role of mutations in different Ras family members (such as N-Ras) is rapidly emerging as an essential tool for patient selection in this disease [5].

NSCLC in women is a growing health concern in Western countries [6]. Gender-related factors (modulating smoking-dependent and independent carcinogenesis) may contribute to this increased incidence [7]. The disease is reported as a peculiar pathological and molecular entity: in particular, women are more often affected by lung adenocarcinoma and the tumor arises among never- or light-smokers with higher frequency than in men [7].

Following the observation that female gender was one of the clinical features associated with a benefit from anti-EGFR tyrosine-kinase inhibitors (TKIs) (together with Asian origin, adenocarcinoma histology and smoking habits) [8,9], deeper insights into the EGFR biology allowed to identify somatic mutations in the kinase domain of the EGFR gene [10]. Nowadays, EGFR mutational status is a well-recognized predictive factor for anti-EGFR TKIs activity and an essential tool for treatment allocation in advanced lung adenocarcinoma [11].

However, the impact of first-line platinum-based chemotherapy (CT) in female patients with EGFR mutated adenocarcinoma is a matter of debate and no definitive data emerge from subgroup analyses of randomized trials comparing CT with TKIs in the first-line setting [8]. The results of a recent meta-analysis of prospective and retrospective studies suggest that EGFR mutations may be associated with higher response rate (RR) to CT, but may not predict the benefit of CT with regard to progression-free survival (PFS) and overall survival (OS) [12].

Among the other molecular aberrations involved in NSCLC progression, K-Ras is certainly one of the most extensively studied [13]: K-Ras mutations (mainly located in codons 12 and 13 of exon 2) are reported in up to 30% of NSCLC cases [13,14], especially among smokers. It is worth noting that EGFR and K-Ras mutations are rarely found in the same tumor, suggesting that they may drive functionally different carcinogenetic processes [13,14]. Direct targeting of K-Ras has recently raised some concerns, as this represents a key transduction pathway in both normal and tumor tissues. Moreover, several parallel escape mechanisms have been identified [15]. Moving from these considerations, alternative targeting of K-Ras is currently under evaluation, e.g. by inhibiting BRAF or MEK activity [15].

Inconclusive evidence exists about the role of K-Ras mutations in predicting the benefit of CT in lung adenocarcinoma [16]. On the other hand, retrospective subgroup analyses seem to suggest that patients with K-Ras mutated tumors show a primary resistance to anti-EGFR TKIs [14]. Two meta-analyses demonstrated that the objective RR to anti-EGFR TKIs in K-Ras mutated tumors is inferior to wild-type ones [17,18]. However, no definitive conclusion was reported in terms of PFS and OS.

In our clinically enriched series (Caucasian women with lung adenocarcinoma) we aimed at investigating the possible predictive significance of EGFR and K-Ras mutations with respect to both first-line platinum-based CT and salvage treatment with the anti-EGFR TKI erlotinib.

## Methods

### Eligibility criteria

We retrospectively identified consecutive women with histologically or cytologically confirmed stage IIIB-IV adenocarcinoma of the lung treated at five Italian Institutions between 2007 and 2010. All patients had their tumor samples available for centralized molecular determinations and were included in the descriptive analyses of mutation frequency. Patients who had received any first-line platinum-based combination regimen for advanced disease and had undergone tumor evaluations were included in the correlative analyses for response and survival.

Pre-treatment evaluation included: clinical history, physical examination, complete blood cell count and biochemistry, computed tomography scan of the chest and computed tomography scan or ultrasonography of the abdomen. Patients with central nervous system (CNS) metastases were considered eligible if they were asymptomatic or stable at imaging evaluation performed 1 month after the completion of brain radiotherapy.

### Treatment

Any platinum-containing regimen was allowed as upfront therapy: CT was administered for a maximum of 6 courses in responding patients and no maintenance treatment was administered. Salvage treatment with erlotinib 150 mg/die orally was given on the basis of clinician’s choice according to initial marketing indications of this drug: this strategy was not pre-planned. The choice between erlotinib and CT was driven by clinical factors (i.e. performance status, organ function, life expectancy, tolerance and response to previous CT). Best supportive care alone was offered to patients with reduced life expectancy (<4 weeks), compromised liver, kidney or bone marrow function, and deteriorated performance status.

Anti-EGFR TKI treatment was administered until progression, unacceptable toxicity or patient refusal. Re-staging during treatment was planned every 12 weeks and objective responses were evaluated according to the Response Evaluation Criteria for Solid Tumors (RECIST) v. 1.0 [19]. All the responses and progressions were retrospectively evaluated by the investigators and no independent revision was performed.

Written informed consent for treatment, molecular analyses and data collection was obtained from each patient according to local Institution policies.

The Ethics Committee of Area Vasta Nord--‐Ovest approved the study.

### Mutational analysis

#### Microdissection and DNA extraction

Serial 5-μm sections were taken from formalin fixed paraffin embedded (FFPE) tissues. The last section was stained with hematoxilin-eosin (H&E), the tumor area was marked and the percentage of tumor cells was estimated by a pathologist. The tumor tissue was manually microdissected from one to three unstained sections previously submitted to xylene deparaffination, and was lysed overnight at 56°C in 180 μl of ATL buffer and 20 μl of proteinase K (QIAGEN, Hilden, Germany). DNA was purified using the spin column procedure (QIAamp minikit; QIAGEN) and finally reconstituted in 40 μl of AE buffer. DNA content was measured with a Nanodrop-1000 spectrophotometer (Thermo Fisher Scientific Inc. Waltham, MA) and was kept at 4°C before use.

#### Detection of EGFR mutations by PCR single-strand conformation polymorphism (SSCP) and direct DNA sequencing

EGFR mutation analysis was performed by amplifying exons 18 to 21 as follows. 5 μL of genomic DNA concentrated 20 ng/μL was amplified in a 25-μl PCR mixture containing 12.5 μL of HotStarTaq Master Mix Kit (QIAGEN) and 0.5 μL of 20 uM forward and reverse primers. All reactions were done in duplicate. The following PCR primers for EGFR exons 18–21 were used:

- for exon 18, 5′(Fw)-CTCTGTGTTCTTGTCCCCCC-3′
- and 5′(Rev)-GCCTGTGCCAGGGACCTTAC-3′ (amplicon size, 166 bp);
- for exon 19, 5′(Fw)-CATGTGGCACCATCTCACA-3′
- and 5′(Rev)-CCACACAGCAAAGCAGAAAC-3′ (amplicon size, 179 bp);
- for exon 20, 5′(Fw)-CACACTGACGTGCCTCTCC-3′
- and 5′(Rev)-TATCTCCCCTCCCCGTATCT-3′ (amplicon size, 250 bp);
- for exon 21, 5′(Fw)-CCTCACAGCAGGGTCTTCTC-3′
- and 5′(Rev)-AATGCTGGCTGACCTAAAGC-3′ (amplicon size, 215 bp).

A mock control with no addition DNA was processed in parallel with each sample. Cycling conditions were as follows: initial denaturation (95°C, 15 min), then 40 cycles of denaturation (95°C for 30 sec), annealing (58°C for 60 sec) and synthesis (72°C for 60 sec). All PCR products were visualized by electrophoresis on a 1.5% agarose gel stained with ethidium bromide. All PCR products were then diluted 1:1 with denaturing solution (1% xylene cyanol, 1% bromophenol blue, 0.1 mM EDTA and 99% formamide), denatured at 95°C for 5 min and immediately placed on ice to prevent annealing of the single-strand products. SSCP was carried out on a GenePhor Electrophoresis Unit using GeneGel Excel 12.5/24 (12.5% T, 2% C) (GE Healthcare Biosciences AB, Uppsala, Sweden), according to the manufacturer’s instructions. Electrophoresis (600 V, 25 mA, 15 W) was performed at 18°C for 100 min. Gels were stained with the DNA Silver Staining Kit (GE Healthcare Biosciences AB), according to the manufacturer’s instructions. Altered migrations patterns in two independent PCR-SSCP runs were indicative of DNA mutations. All PCR products were purified with QIAquick PCR Purification kit (QIAGEN) and labeled using the BigDye Terminator (version 3.1) Cycle Sequencing Kit (Applied Biosystems, Foster City, CA, USA) according to the manufacturer’s protocol, and were followed by sequencing in an ABI 3130 Genetic Analyzer (Applied Biosystems). Both forward and reverse sequencing reactions were performed with the same PCR primers at 0.8 μmol/L in a final volume of 20 μL. The sequencing data were visualized using of Sequencing Analysis (Applied Biosystems), and were independently evaluated by two investigators.

#### Detection of K-Ras mutation by pyrosequencing

The K-Ras codons 12/13 mutational analysis was evaluated amplifying 5 μL of genomic DNA concentrated 20 ng/μL by Anti-EGFR MoAb response® (KRAS status) CE-IVD marked kits (DIATECH Pharmacogenetics, Italy) on Rotor-GeneTM 6000 (Corbett Research, Australia) following the manufacturer’s instructions. The resulting PCR product was immobilized onto magnetic streptavidin-coated beads via the biotin/streptavidin interaction. The bead/DNA complex was then washed and added to 1.65 pmol of pyrosequencing primer included in the same kit. The primed single-stranded DNA templates were then transferred to the microtitre plate-based PSQ HS 96 A Pyrosequencer (Biotage, Sweden), where real-time sequencing of the sequence surrounding codon 12/13 exon 2 of K-Ras was performed by using PyroMark Gold Q96 reagents (QIAGEN) on PyroMarkTM Q96 ID instrument (Biotage). The results were analyzed using PyroMark Q24 1.0.9 software, and were independently evaluated by two investigators.

### Statistical analysis

EGFR and K-Ras mutations were correlated with clinical features such as smoking habits (never or former vs. current smokers) and brain metastases (yes vs. no) by means of the chi-square test.

The primary endpoints for correlative analyses were PFS times after first-line CT and salvage treatment with erlotinib. RR and OS were secondary endpoints.

PFS was calculated from the start of first-line CT or salvage erlotinib to the date of disease progression or death (whichever occurred first). OS was calculated from the start of first-line CT or salvage erlotinib to the date of death or last follow up visit. PFS and OS were estimated using the Kaplan-Meier method. Differences across curves were analyzed by log-rank test, while correlation with RR was estimated using the chi-square test. Statistical significance was set at p < 0.05 for a two-tailed test. Graphpad Prism 6 software was used for statistical analysis.

## Results

### Patient characteristics

A total of 103 women were identified and included in the descriptive analysis. Sixty-six patients, who received first-line platinum-based CT and whose clinical and molecular data were available, were included in the correlative analysis. The main characteristics are summarized in Table 1.

**Table 1:** **Patient demographic and clinical characteristics**

	**Descriptive cohort (n = 103)**		**Correlative cohort (n = 66)**		**Anti-EGFR treated (n = 39)**	
	**n**	**%**	**n**	**%**	**n**	**%**
Age (years)						
Median	64		60		62	
Range	38-88		38-81		38-77	
Smoking history						
Never smokers	63	61	40	60	27	69
Former smokers	8	8	5	8	4	10
Current smokers	32	31	21	32	8	21
Stage						
IIIB	30	29	17	26	11	28
IV	73	71	49	74	28	72
Number of sites of metastases						
1	16	16	18	27	9	23
2	25	24	26	39	13	33
≥3	62	60	22	34	17	44
CNS metastases						
Yes	14	14	12	18	7	18
No	89	86	54	82	32	82
Previous surgery on primary						
Yes	46	45	20	30	14	36
No	57	55	46	70	25	64
Previous (neo-)adjuvant CT						
Yes	9	9	5	8	4	10
No	94	91	61	92	35	90
Previous adjuvant RT						
Yes	4	4	2	4	3	8
No	99	96	63	96	36	92
First-line CT regimen						
Platinum + Paclitaxel	10	10	10	15	5	13
Platinum + Gemcitabine	39	39	39	59	21	54
Platinum + Pemetrexed	13	13	13	20	6	15
Cisplatin + Vinorelbine	2	2	2	3	1	3
Cisplatin-based triplet CT	2	2	2	3	2	5
Single-agent CT*	26	26	-	-	4	10
Best supportive care only	8	8	-	-	-	-
Anti-EGFR TKI treatment line						
2	28	27	24	36	27	69
≥3	12	12	9	14	12	31

*Abbreviations*: *TKIs* tyrosine-kinase inhibitors, *ECOG* Eastern Cooperative Oncology Group, *CNS* central nervous system, *CT* chemotherapy, *RT* radiotherapy. *Single agent platinum, gemcitabine, vinorelbine, pemetrexed.

### EGFR and K-Ras mutations: correlation with clinical variables

EGFR and K-Ras mutations were found in 30% and 13% of the cases, respectively (Table 2): in this series mutations in EGFR and K-Ras were mutually exclusive. EGFR mutations were exon 19 deletion (77%), exon 21 L858R mutation (13%) and exon 20 mutation (10%). Among the K-Ras mutant cases, 77% and 23% had mutations in codons 12 and 13, respectively.

**Table 2:** **EGFR and K-Ras mutational status**

	**Descriptive cohort (n = 103)**		**Correlative cohort (n = 66)**		**Anti-EGFR treated (n = 39)**	
	**n**	**%**	**n**	**%**	**n**	**%**
EGFR mutational status						
Wild-type	72	70	47	71	24	62
Mutant	31	30	19	29	15	38
Sites of EGFR mutation						
Exon 19 deletion	24	77*	14	74*	12	80*
E746_A750del	19	62*	10	54*	8	52*
E746_R748del	1	3*	1	5*	-	-
E746_E749 > Y	1	3*	1	5*	1	7*
L747_A750 > P	1	3*	1	5*	1	7*
L747_T751del	1	3*	1	5*	1	7*
S751_I759del	1	3*	-	-	1	7*
Exon 20 mutation	3	10*	2	10*	-	-
G796D	2	7*	1	5*	-	-
A767_V769dup	1	3*	1	5*	-	-
Exon 21 mutation	4	13*	3	16*	3	20*
L858R	4	13*	3	16*	3	20*
K-Ras mutational status						
Wild-type	76	73	46	70	30	77
Mutant	13	13	9	14	4	10
Not evaluable	14	14	11	16	5	13
Sites of K-Ras mutation						
Codon 12 mutation	10	77*	7	78*	4	100*
G12C	6	46*	4	44*	3	75*
G12D	1	8*	-	-	-	-
G12V	3	23*	3	34*	1	25*
Codon 13 mutation	3	23*	2	22*	-	-
G13D	3	23*	2	22*	-	-

*Calculated as % of total EGFR and K-Ras mutations in each patient cohort.

K-Ras mutations were significantly associated with smoking history: indeed, mutations were more frequent in current smokers compared to former or never smokers (28% vs. 8%; p = 0.024). On the contrary, EGFR mutations occurred more frequently in never smokers, although the difference did not reach statistical significance (35% vs. 19%; p = 0.108).

In our series, there was no correlation between EGFR (mutant vs. wild-type: 13% vs. 15%; p = 1.000) or K-Ras (mutant vs. wild-type: 0% vs. 18%; p = 0.208) status and the development of CNS metastasis during the clinical course of the disease.

At a median follow up of 46.7 months, 97% of the patients had progressed after first-line CT and 55% had died.

### EGFR and K-Ras mutations: correlation with benefit from first-line CT

**Figure 1:**
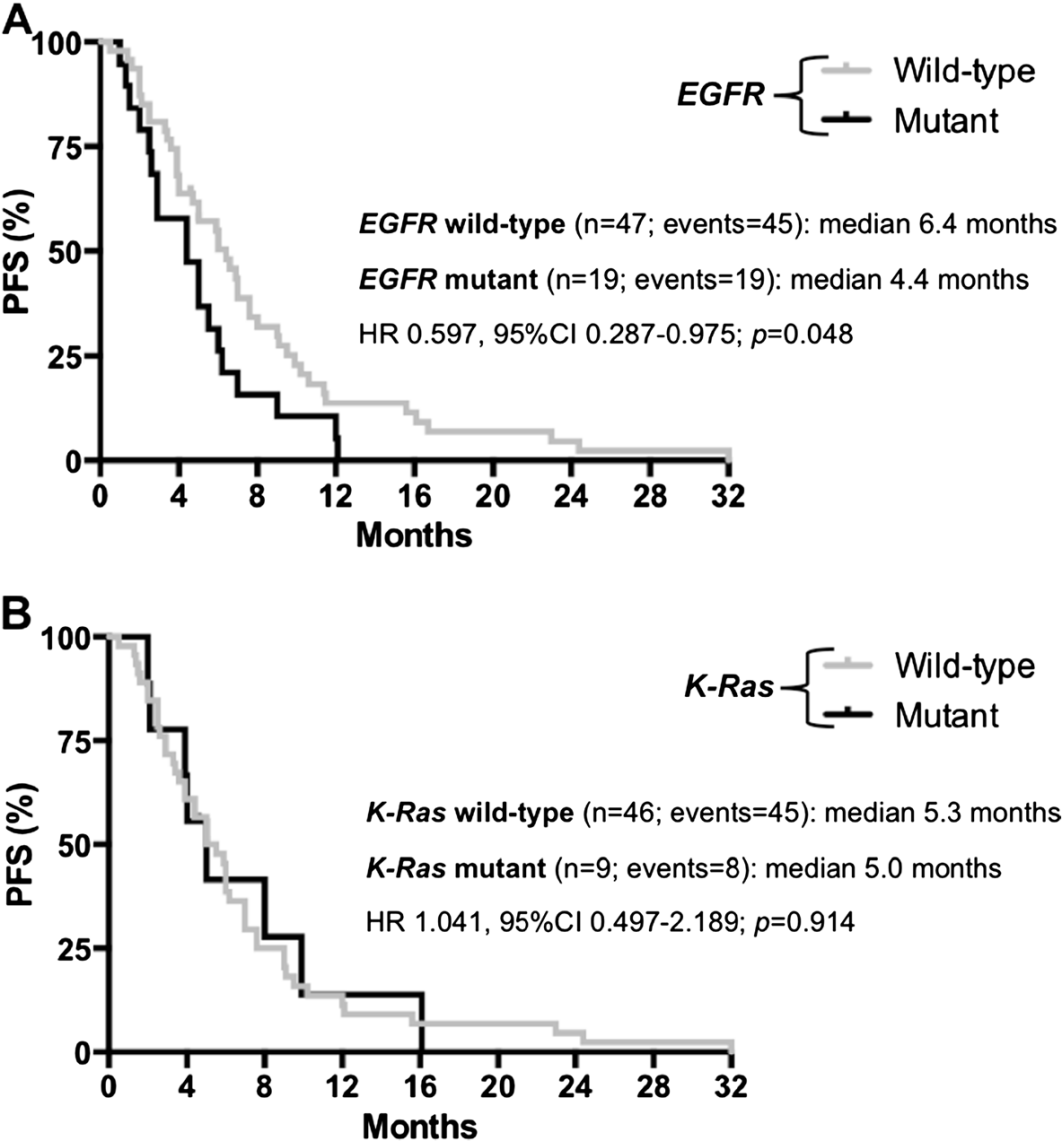
**Progression--‐free survival (PFS) with CT according to molecular parameters. A**. Progression-free survival (PFS) according to EGFR status among patients treated with first-line platinum-based combinations. **B**. Progression-free survival (PFS) according to K-Ras status among patients treated with first-line platinum-based combinations.

Sixty-six patients were treated with first-line platinum-containing CT. When the EGFR mutational status was considered, a significantly longer median PFS was observed among women with wild-type tumors than among women harboring mutant tumors (6.4 vs. 4.4 months, respectively; HR 0.597, 95% CI 0.287-0.975; p = 0.048) (Figure 1A). As regards K-Ras status, no significant difference was observed between wild-type and mutant patients in terms of median PFS (5.3 vs. 5.0 months, respectively; HR 1.041, 95% CI 0.497-2.189; p = 0.914) (Figure 1B). When additional prognostic factors were analyzed (age, smoking habits, stage of disease, type of EGFR mutation), none was significantly associated with PFS (Table 3).

**Table 3:** **Univariate analysis for PFS**

	**Correlative cohort (n = 66)**		**Anti-EGFR treated (n = 39)**	
	**Median**	**p**	**Median**	**p**
Smoking history		0.246		0.238
Never smokers	5.0		3.2	
Former smokers	5.0		5.6	
Current smokers	7.0		10.0	
Stage		0.130		0.423
IIIB	7.6		11.4	
IV	5.0		4.8	
Age		0.233		0.894
<70 years	5.5		7.2	
≥70 years	6.5		5.0	
EGFR mutation*		0.373		0.052
Exon 19 mutation	4.4		12.7	
Exon 20 mutation	8.0		NA	
Exon 21 mutation	3.2		1.4	

*Abbreviations*: *PFS* progression-free survival, *NA* not applicable. *Analysis is restricted to EGFR mutant patients only.

As regards RR, 26 (40%) achieved an objective response, 20 (30%) reported disease stabilization and 20 (30%) progressed during CT. Neither EGFR nor K-Ras mutations significantly correlated with response to first-line CT: RR was 32% vs. 43% in EGFR mutant vs. EGFR wild-type tumors (p = 0.579), and 33% vs. 39% in K-Ras mutant vs. K-Ras wild-type tumors (p = 1.000), respectively. No statistically significant differences in median OS were observed according to EGFR mutational status (wild-type vs. mutant: 29.5 vs. 26.2 months; HR 0.967, 95% CI 0.473-1.974; p = 0.926) or K-Ras (wild-type vs. mutant: 26.1 vs. 26.8 months; HR 0.877, 95% CI 0.342-2.207; p = 0.771).

### EGFR and K-Ras mutations: correlation with benefit from salvage erlotinib

Thirty-nine (59%) women received salvage treatment with erlotinib. At the time of the analysis, 74% progressed and median PFS for the entire population was 7.2 months. We observed a trend toward a higher benefit from erlotinib when administered in second line compared to third or fourth line (10.0 vs. 4.0 months, respectively; HR 0.587, 95% CI 0.206-1.145; p = 0.132): this difference was less evident when the analysis was restricted to EGFR mutant patients (12 and 3 patients for second-line and ≥ third-line treatment, respectively: 11.4 vs. 24.2 months). Unfortunately, five patients with EGFR mutant tumors experienced rapid worsening of general conditions after first-line CT and died before having received any anti-EGFR TKI. None of the investigated clinical features was significantly associated with PFS in patient treated with erlotinib (Table 3).

**Figure 2:**
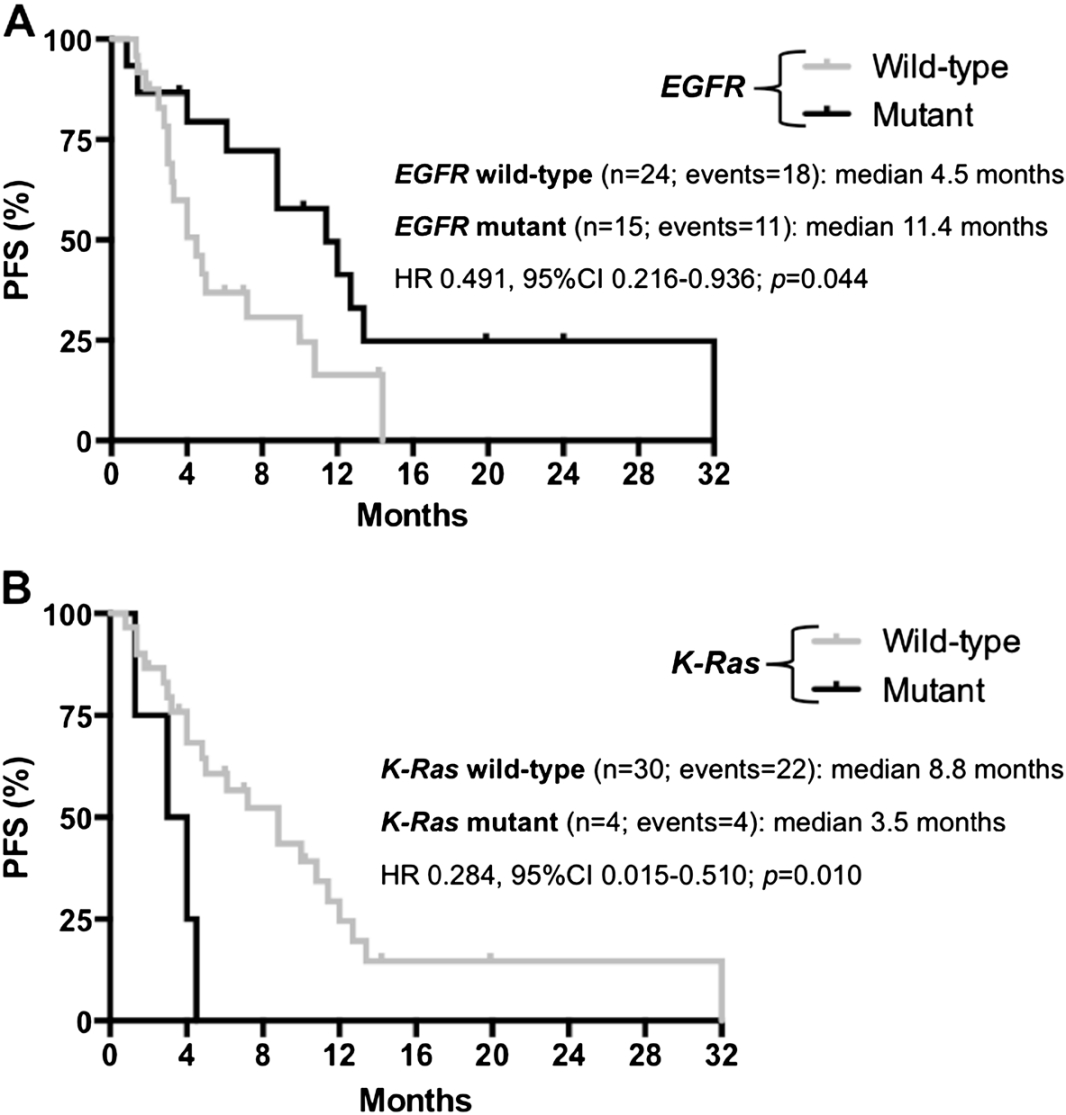
**Progression--‐free survival (PFS) with anti--‐EGFR TKI according to molecular parameters. A**. Progression-free survival (PFS) according to EGFR status among patients treated with anti-EGFR TKI. **B**. Progression-free survival (PFS) according to K-Ras status among patients treated with anti-EGFR TKI.

EGFR mutations were significantly correlated with RR to salvage anti-EGFR TKI (mutant vs. wild-type: 60% vs. 12.5%; p = 0.004) and longer median PFS (mutant vs. wild-type: 11.4 vs. 4.5 months; HR 0.491, 95% CI 0.216-0.936; p = 0.044) (Figure 2A). There was a trend toward improved OS among mutant patients compared to wild-type ones (21.0 vs. 9.2 months, respectively; HR 0.560, 95% CI 0.231-1.156; p = 0.131).

PFS of K-Ras mutant women treated with salvage anti-EGFR TKI was significantly reduced compared to that of wild-type ones receiving the same drug (median: 3.5 vs. 8.8 months; HR 0.284, 95% CI 0.015-0.510; p = 0.010) (Figure 2B). None of the patients harboring a K-Ras mutant tumor achieved an objective response to erlotinib compared with 10 out of 30 wild-type patients evaluable for the analysis (RR: 0% vs. 33%; p = 0.296): of these 10 K-Ras wild-type patients, 8 harbored an EGFR mutant tumor. Of the two groups identified by K-Ras analysis, significantly shorter OS results were also reported in the mutant subset (median: 3.9 vs. 19.8 months in wild-type patients; HR 0.158, 95% CI 0.001-0.075; p < 0.001).

## Discussion

We retrospectively evaluated the outcomes of Caucasian women with lung adenocarcinoma, undergoing first-line platinum-based CT and subsequent salvage anti-EGFR TKI therapy, in relation to EGFR and K-Ras mutational status.

The clinical selection we applied (i.e. by gender and histology), together with the high percentage of never smokers in our series, is probably responsible for the high incidence of EGFR mutations (30%) and the low frequency of K-Ras mutations (13%) observed. As expected, K-Ras mutations were more frequent in current smokers, while a formally significant influence of smoking habits on the EGFR mutation rate was not detected: this was probably due to the small sample size and the potential confounding effect of other clinical selection criteria, such as gender and histology. In our series no patient had concomitant EGFR and K-Ras mutations.

Patients received first-line platinum-based CT according to clinical characteristics and physicians’ choice. Retrospectively, the correlation between clinical parameters (RR, PFS and OS) and EGFR and K-Ras mutational status showed that women with EGFR mutant tumor achieved a marginally lower RR to first-line platinum-based combination (32% vs. 43%; p = 0.579) and a significantly shorter PFS (4.4 vs. 6.4 months; HR 0.597; p = 0.048) compared to those with wild-type disease. There was no impact of the type of EGFR mutations on the PFS results and, as observed by previous reports, K-Ras mutations did not play a role in predicting the benefit from standard CT [16]. The effect of EGFR status on the efficacy of first-line platinum-based CT has been only rarely reported in the majority of studies and always with conflicting results [12]. The most recent and reliable data may be derived from trials of first-line CT vs. anti-EGFR TKIs or CT plus a third targeted agent in molecularly unselected patients [8]. However, none of the reported analyses demonstrated a significant impact of EGFR mutational status on the benefit from first-line platinum-containing CT. Looking at the published or presented results, most trials suggest no role or, at best, slightly better outcome for patients with EGFR mutant tumor when treated with first-line CT [8,12]. However, this remains an open issue and a definitive answer cannot be given since it would, in any case, suffer from several limitations (e.g., no gender selection, retrospective and unplanned nature of the analyses). Our series included only women with lung adenocarcinoma: female gender is an established good prognostic factor in NSCLC independently of disease stage, molecular status and treatment [20] and we cannot exclude a potential synergistic effect of gender and EGFR mutational status on the results we reported. Clinical enrichment in our study could also be the cause of the interesting median OS we observed (up to 29.5 months), not always reached in a population of unselected advanced NSCLC patients. However, although limited in size, our results further confirm the need of an upfront molecular characterization of all patients with lung adenocarcinoma before starting first-line treatment. In our series 5 patients with EGFR mutant disease died without receiving any anti-EGFR treatment, mainly because of a rapid worsening of performance status due to disease progression after first-line CT. Considering the huge amount of data supporting the superiority of anti-EGFR TKIs compared with CT in these patients, every effort should be made to clarify the molecular profile of a lung adenocarcinoma before starting any treatment in everyday clinical practice.

Thirty-nine women received salvage erlotinib irrespectively of EGFR or K-Ras mutational status. EGFR mutations are a recognized predictive biomarker [11] and a regulatory requirement for the use of an anti-EGFR TKI as first-line approach [21]. Therefore, it is not surprising that, in our series, the benefit from erlotinib administered at progression was restricted to EGFR mutant cases both in terms of RR (p = 0.004) and PFS (HR 0.491; p = 0.044). Moreover, women with EGFR mutant tumor treated with front-line CT and salvage anti-EGFR TKI showed a trend toward a better OS in comparison with those affected by EGFR wild-type tumor undergoing the same sequence (HR 0.560; p = 0.131). We might speculate that the disadvantage in PFS obtained with first-line CT in EGFR mutated women can be counterbalanced by the better results achieved with salvage anti-EGFR TKI. However, since a significant proportion of women with EGFR mutant tumors received no anti-EGFR TKI after first-line CT, once again we believe that these results stress the importance of a pre-specified sequence of approaches (upfront TKI followed by salvage CT) in women harboring molecularly-addicted tumors in order to achieve the best outcome. The lack of any impact of the type of EGFR mutation on PFS could be due, at least in part, to the limited number of cases in our series.

In our clinically enriched population K-Ras mutations were not predictive of benefit from CT. As a matter of fact no difference in RR (33% vs. 39%) and PFS (5.0 vs. 5.3 months) was observed between patients with K-Ras mutant tumor and those with wild-type disease. The most recent report in a similar setting (i.e. EGFR wild-type patients treated with erlotinib after progression to first-line CT) was the retrospective evaluation of K-Ras status in the TAILOR study [22]. In this randomized phase III trial K-Ras mutations were neither prognostic nor predictive of benefit from docetaxel and no significant interaction was demonstrated between K-Ras status and PFS or OS.

On the contrary, our results seem to suggest a putative negative predictive significance of K-Ras mutations for anti-EGFR TKI activity. None of the patients with K-Ras mutant tumor achieved an objective response with salvage erlotinib and these patients experienced a significantly worse median PFS (HR 0.284; p = 0.010) and OS (HR 0.158; p < 0.001) compared with patients affected by wild-type NSCLC. Considering that the benefit from CT did not differ between the two groups and that K-Ras mutated women experienced not only a significantly shorter PFS on TKI but, in particular, a significantly reduced OS, we could argue that TKI administration in such a population could be potentially detrimental. However the limited number of patients in our series prevents us from drawing any definitive conclusion. Other larger data sets did not suggest any relevance of K-Ras mutations in determining the benefit of CT [16], and taking into account the above mentioned conflicting results about the predictive role for anti-EGFR TKIs [17,18], we do not believe that routine K-Ras sequencing should be considered in all patients with lung adenocarcinoma outside clinical and translational trials. This is particularly relevant when the tumor is not easily accessible for biologic sample collection and thus oncologists need to spare tissue for fundamental analyses, such as EGFR mutations and EML4-ALK translocation.

## Conclusions

In our series of Caucasian women with advanced adenocarcinoma of the lung EGFR mutations seem to be associated with lower benefit from first-line platinum-based CT. If confirmed in larger, prospectively collected, datasets these results may help to shed light on the potential influence of gender on EGFR biology in NSCLC. We confirmed that the presence of an EGFR mutation strongly predicts the benefit of salvage treatment with erlotinib, stressing the role of this drug as first-line option in molecularly selected patients. The role of K-Ras mutations as biomarkers of resistance to anti-EGFR TKIs in NSCLC is still unclear and surely deserves further investigations.

## Abbreviations

CNS: Central nervous system
CT: Chemotherapy
EGFR: Epidermal growth factor receptor (gene)
EML4-ALK: echinoderm microtubule-associated protein-like 4 (EML4) - anaplastic lymphoma kinase (ALK) (genes)
K-Ras: V-KI-RAS2 Kirsten rat sarcoma viral oncogene homolog (gene)
NSCLC: Non-small cell lung cancer
OS: Overall survival
PFS: Progression-free survival
RECIST: Response evaluation criteria for solid tumors
RR: Response rate
TKI(s): Tyrosine-kinase inhibitor(s)
